# Multimodal Assessment of Tissue Response After Thyroid Radiofrequency Ablation: Clinical, Imaging, Histopathological and Molecular Insights

**DOI:** 10.3390/jcm15156031

**Published:** 2026-08-03

**Authors:** Domenico Parmeggiani, Gerardo Amabile, Alessio Cece, Sergio Surfaro, Giancarlo Moccia, Francesco Miele, Pasquale Luongo, Manuela Miccio, Rossella Sperlongano, Agostino Fernicola, Paola Della Monica, Federica Colapietra, Marina Di Domenico, Eduardo Clery, Massimo Agresti, Renato Franco

**Affiliations:** 1 Department of Integrated Activities in Surgery, Orthopedy and Hepato-Gastroenterology, Universitary Policlinico “Luigi Vanvitelli”, 80138 Naples, Italy; alessio.cece@unicampania.it (A.C.); giancarlo.moccia@policliniconapoli.it (G.M.); francesco.miele@unicampania.it (F.M.); pasquale.luongo@unicampania.it (P.L.); manuela.miccio@studenti.unicampania.it (M.M.); massimo.agresti@unicampania.it (M.A.); 2 Casa Di Cura Ospedale Internazionale—NefroCenter, 80121 Naples, Italy; gerardoamabile@dr.com; 3 Medibit SRL, 80123 Naples, Italy; medibit@libero.it; 4 Department of Precision Medicine, University of Campania “Luigi Vanvitelli”, 80138 Naples, Italy; rossella.sperlongano@unicampania.it (R.S.); paola.dellamonica@unicampania.it (P.D.M.); federica.colapietra@unicampania.it (F.C.); marina.didomenico@unicampania.it (M.D.D.); 5 Operative Unit of General and Emergency Surgery, Department of Advanced Biomedical Sciences, University of Naples Federico II, 80131 Naples, Italy; agostinofernicola@yahoo.it; 6 Pathology Unit, Departement of Mental and Physical Health and Preventive Medicine, University of Campania “Luigi Vanvitelli”, 80138 Naples, Italy; eduardo.clery@unicampania.it (E.C.); renato.franco@unicampania.it (R.F.)

**Keywords:** thyroid nodules, radiofrequency ablation, translational medicine, tissue-level characterization, extracellular vesicles, miRNA, 3D imaging, intelligent multimodal platform

## Abstract

**Background:** Ultrasound-guided radiofrequency ablation (RFA) has become an established minimally invasive treatment for selected benign and autonomously functioning thyroid nodules. Although clinical outcomes are well documented, the biological mechanisms underlying tissue remodeling after ablation remain incompletely characterized. The integration of imaging, molecular, and histopathological data may provide a more comprehensive understanding of treatment response. **Methods:** We conducted a prospective monocentric observational translational cohort study including 60 patients undergoing ultrasound-guided RFA for benign or autonomously functioning thyroid nodules between November 2024 and December 2025. Patients underwent standardized pre-procedural assessment including high-resolution ultrasound with volumetric evaluation, three-dimensional reconstruction, and collection of peripheral blood samples for exploratory extracellular vesicle (EV)-derived microRNA (miRNA) profiling. Clinical outcomes, volumetric changes, vascular remodeling, and procedural complications were assessed during scheduled follow-up visits (mean follow-up duration: 11 months; range: 5–17 months). A subgroup of patients who subsequently required surgery because of insufficient volumetric response underwent thyroidectomy, allowing histopathological evaluation of post-ablation tissue changes. Clinical, imaging, molecular, and pathological data were integrated within a multimodal translational framework. **Results:** RFA resulted in a mean nodule volume reduction of 56% at early follow-up, with progressive volumetric reduction observed during longitudinal ultrasound evaluation. A clinically relevant improvement in compressive symptoms was observed, with a 44% reduction in symptom burden. Doppler ultrasound demonstrated progressive changes in vascular patterns consistent with post-ablation devascularization and tissue remodeling. Ten patients underwent thyroidectomy after RFA because of insufficient volumetric response, providing histological validation of treatment-induced tissue alterations, including coagulative necrosis, fibrosis, inflammatory changes, and architectural remodeling. Exploratory EV-derived miRNA profiling was incorporated into the translational workflow to investigate potential molecular correlates of treatment response; however, molecular findings require further validation in larger cohorts. **Conclusions:** This study provides a multimodal translational characterization of tissue response after thyroid RFA by integrating clinical outcomes, ultrasound imaging, histopathology, and exploratory EV-derived miRNA analysis. The proposed framework may contribute to a deeper understanding of biological changes following thermal ablation and support future investigations aimed at identifying reliable biomarkers of treatment response.

## 1. Introduction

Ultrasound-guided thermal ablation techniques, particularly radiofrequency ablation (RFA), have emerged over the past two decades as minimally invasive alternatives to surgery for the treatment of benign thyroid nodules. RFA delivers high-frequency alternating current through an electrode inserted into the target lesion under ultrasound guidance, generating thermal energy that induces coagulative necrosis and progressive volume reduction of the treated tissue. This mechanism results in gradual nodule shrinkage while preserving surrounding thyroid parenchyma and adjacent critical structures.

Recent systematic reviews and clinical studies have consistently demonstrated that RFA achieves significant nodule volume reduction, generally ranging between 50% and 90%, with a low complication rate and high patient satisfaction [1,2]. A large body of evidence now supports its safety and efficacy in both benign and autonomously functioning thyroid nodules, positioning RFA as a valid alternative to surgery in appropriately selected patients [3]. Current international guidelines reflect this growing evidence base. The European Thyroid Association recommends image-guided thermal ablation as a treatment option for benign thyroid nodules causing compressive symptoms or cosmetic concerns, as well as for selected autonomously functioning thyroid nodules, particularly in patients who decline or are not candidates for surgery [4]. Similarly, consensus statements and technical recommendations emphasize careful patient selection based on ultrasound characteristics, cytology confirming benignity, and clinical symptom burden [4,5]. However, these indications remain largely based on clinical and imaging criteria, without integration of molecular or tissue-level biomarkers [4].

Despite the well-established clinical efficacy of RFA, its biological mechanisms of action remain incompletely understood. In particular, the relationship between macroscopic imaging changes and underlying histopathological remodeling and molecular responses has not been fully elucidated. Post-ablation tissue is typically not available for analysis unless surgery is subsequently required, limiting systematic evaluation of the biological effects of thermal injury in vivo.

Recent advances in translational medicine have introduced new tools capable of bridging this gap. Three-dimensional (3D) imaging reconstruction enables more precise volumetric and spatial characterization of thyroid nodules before and after treatment. In parallel, extracellular vesicle (EV)-derived microRNAs (miRNAs) have emerged as promising circulating biomarkers reflecting tissue injury, cellular stress, and remodeling processes. Furthermore, the integration of multimodal data through computational platforms offers the opportunity to correlate imaging, molecular, and histopathological information into a unified analytical framework.

In this context, the present study aims to establish a 360° translational framework for thyroid RFA, integrating pre-procedural molecular profiling, advanced imaging reconstruction, and post-ablation tissue characterization in patients undergoing clinical follow-up, including those subsequently requiring surgery. By correlating clinical outcomes with volumetric, vascular, histopathological, and molecular changes, this study seeks to provide a more comprehensive understanding of tissue response to RFA and to support a more personalized and evidence-based approach to patient selection.

As summarized in Table 1, current international guidelines for thyroid RFA are primarily based on clinical, cytological, and imaging criteria, without incorporation of molecular or tissue-level biomarkers, highlighting a persistent gap in patient stratification and biological characterization. In this context, the present study aims to evaluate an exploratory translational framework for thyroid RFA, integrating pre-procedural EV-derived miRNA profiling, advanced imaging reconstruction, and post-ablation tissue characterization. While the molecular and computational components serve as proof-of-concept analyses, this integrated approach seeks to generate hypotheses regarding the biological underpinnings of tissue response following thermal ablation.

## 2. Materials and Methods

### 2.1. Study Design and Patient Cohort

This prospective monocentric observational translational cohort study was conducted at the General Surgery and Oncological Physiopathology Unit of the University Hospital “Luigi Vanvitelli”, Naples, Italy. The study included patients affected by benign or autonomously functioning thyroid nodules who underwent ultrasound-guided radiofrequency ablation (RFA) between November 2024 and December 2025.

A total of 60 patients were enrolled (48 females and 12 males), with a mean baseline nodule volume of 39.8 mL. Fifteen patients presented autonomously functioning thyroid nodules, defined by suppressed thyroid-stimulating hormone (TSH) levels (<0.4 mIU/L).

Patients were selected according to established indications for thyroid thermal ablation, including symptomatic benign thyroid nodules causing compressive symptoms or cosmetic concerns, autonomously functioning nodules with inadequate response or intolerance to medical therapy, and patients with increased surgical risk or preference for a minimally invasive approach, according to international recommendations [6,7,8,9,10].

Inclusion criteria were:

- Adult patients (≥18 years);
- Benign thyroid nodules confirmed by cytological assessment according to international recommendations;
- Autonomously functioning thyroid nodules eligible for minimally invasive treatment;
- Presence of compressive symptoms, cosmetic concerns, or functional impairment representing an indication for intervention.

Exclusion criteria included:

- Suspicious or malignant cytological findings;
- Previous thyroid surgery;
- Inability to complete clinical follow-up;
- Pregnancy;
- Coagulation disorders or contraindications to percutaneous procedures.

All patients underwent structured clinical and ultrasound follow-up at 1, 3, 6, and 12 months after RFA whenever available. Mean follow-up duration was 11 months (range: 5–17 months). Clinical outcomes, volumetric changes, vascular remodeling, and procedure-related complications were recorded.

The study was conducted in accordance with the Declaration of Helsinki and was approved by the institutional Ethics Committee of the University of Campania “Luigi Vanvitelli”. Written informed consent was obtained from all participants.

During follow-up, a subgroup of patients who showed insufficient volumetric response after RFA underwent thyroidectomy according to clinical judgement and institutional practice. These patients represented a surgical validation subgroup, allowing histopathological characterization of post-ablation tissue remodeling. Overall, 10 patients underwent thyroidectomy after RFA.

#### Endpoints of the Study

Primary Endpoint: The primary clinical outcome was the Volume Reduction Rate (VRR, %) of treated thyroid nodules evaluated at 6 months post-RFA compared with baseline, alongside clinical success (defined as a VRR >/= 50% accompanied by significant symptom relief). Secondary Exploratory Endpoint: Long-term volumetric shrinkage and qualitative/quantitative vascular pattern changes assessed via Color Doppler ultrasound (Lagalla classification) at 1, 3, 6, and 12 months. Improvement in patient-reported compressive symptoms and cosmetic score. Safety profile, including procedural complications and adverse events. Histopathological characterization of post-ablation tissue remodeling in the subgroup of patients subsequently undergoing thyroidectomy. Exploratory profiling of baseline circulating EV-derived miRNAs and initial proof-of-concept multimodal data integration for outcome stratification.

### 2.2. Clinical, Imaging and Procedural Workflow

All patients underwent a standardized pre-procedural evaluation including comprehensive clinical assessment, biochemical evaluation, and high-resolution ultrasound examination. Clinical parameters included compressive symptoms, cosmetic concerns, thyroid function tests, and the presence of autonomously functioning thyroid nodules.

Ultrasound examinations were performed using a Philips Affiniti 50 ultrasound system (Philips Healthcare, Bothell, WA, USA) equipped with a high-frequency linear eL18-4 transducer (4–18 MHz). The system was used for conventional B-mode imaging, color Doppler evaluation, and volumetric assessment of thyroid nodules. Ultrasound acquisition was performed according to a standardized institutional protocol for thyroid imaging.

All ultrasound examinations were performed by a single operator (G.A.) with expertise in thyroid ultrasonography and ultrasound-guided interventional procedures. Longitudinal and transverse images were acquired for each thyroid nodule, allowing evaluation of morphological characteristics, anatomical relationships with surrounding structures, and volumetric assessment.

**Figure 1:**
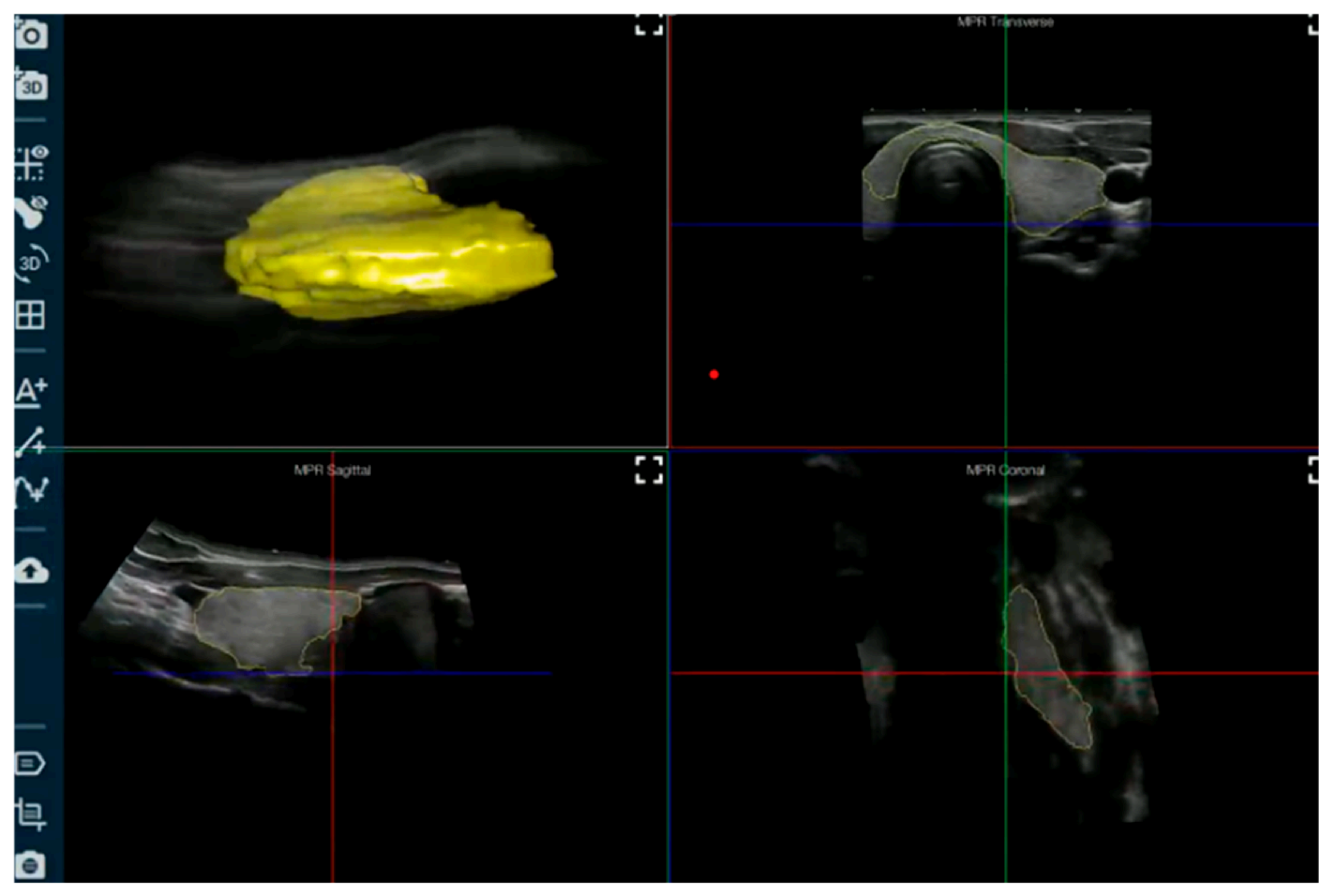
High-resolution ultrasound examination with volumetric analysis and three-dimensional reconstruction of thyroid nodules. Images were obtained from the institutional ultrasound database (AOU Luigi Vanvitelli).

Nodule volume was calculated using the ellipsoid formula (length × width × depth × π/6). Three-dimensional reconstruction was obtained from ultrasound datasets to improve spatial characterization of thyroid nodules and to provide additional visualization of morphological changes occurring after thermal ablation (Figure 1).

**Figure 2:**
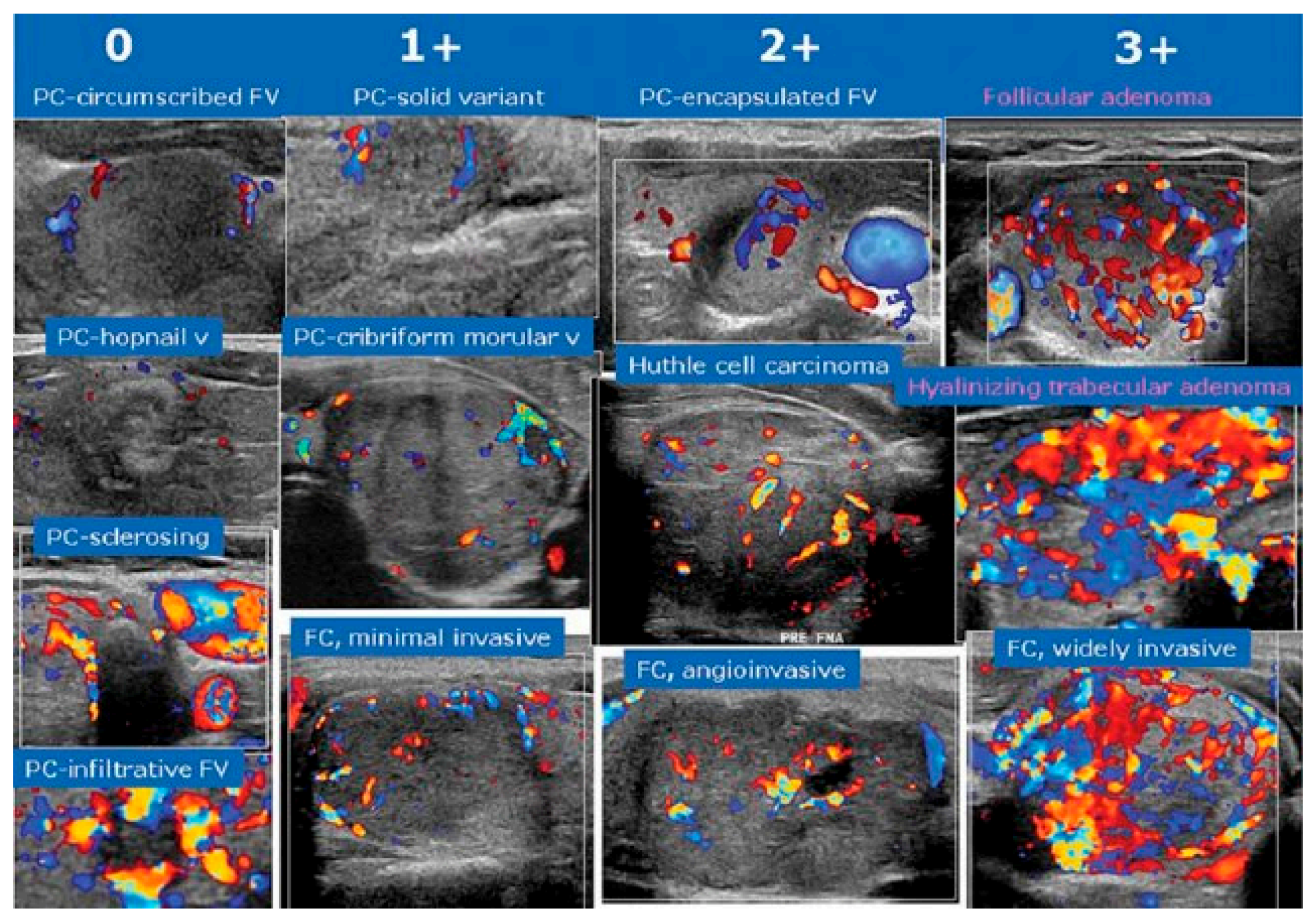
Intranodular vascularity grading on color Doppler and representative tumors. Grade 0: PC, circumscribed FV (1.6 cm); PC, hobnail v (1.4 cm); PC, subcapsular sclerosing FV (1 cm); and PC, infiltrative FV (0.5 cm). Grade 1: PC, solid variant (0.9 cm); PC, cribriform morular v (3.1 cm); and FC, minimally invasive (3 cm). Grade 2: PC, encapsulated FV (1.3 cm); Hürthle cell carcinoma (4.6 cm); and FC, angioinvasive (3.3 cm). Grade 3: follicular adenoma (2.5 cm); hyalinizing trabecular adenoma (2.2 cm); and FC, widely invasive (3.2 cm). FC indicates follicular carcinoma; FV, follicular variant; PC, papillary carcinoma; and v, variant [11].

Vascularization patterns were assessed using the Lagalla classification system, which allows qualitative and semi-quantitative evaluation of intranodular vascularity according to progressive vascular patterns [11]. The vascular pattern was evaluated by the same operator (G.A.) before treatment and during follow-up examinations in order to reduce interobserver variability. Changes in vascular distribution were recorded longitudinally as an indicator of post-ablation tissue remodeling (Figure 2).

Ultrasound follow-up examinations were scheduled at 1, 3, 6, and 12 months after radiofrequency ablation whenever available. During follow-up, nodule volume reduction, vascular pattern modifications, and ultrasound characteristics of the treated area were systematically recorded.

Ultrasound-guided radiofrequency ablation (RFA) was performed according to established international recommendations for thyroid thermal ablation techniques [6,7]. Treatment planning was based on baseline nodule characteristics, including size, location, vascularity, and relationship with adjacent critical structures.

The integration of ultrasound imaging, clinical parameters, extracellular vesicle (EV)-derived microRNA profiling, and histopathological evaluation in selected surgical cases was designed as an exploratory translational workflow aimed at characterizing biological and tissue-level responses following thyroid RFA.

### 2.3. Ultrasound Assessment and Vascular Pattern Analysis

All thyroid nodules were evaluated by standardized high-resolution ultrasound examination before radiofrequency ablation and during scheduled follow-up visits at 1, 3, 6, and 12 months after treatment.

Baseline ultrasound assessment included evaluation of nodule size, volume, echogenicity, margins, internal composition, calcifications, and relationship with adjacent cervical structures. Nodule volume was calculated using the ellipsoid formula (length × width × depth × π/6). Volume reduction rate (VRR) was calculated according to the following formula (1):

VRR (%) = [(initial volume − final volume)/initial volume] × 100.(1)

Three-dimensional reconstruction of ultrasound datasets was performed to improve spatial characterization of thyroid nodules and to provide a volumetric representation of morphological changes occurring after ablation. Three-dimensional reconstructions were generated from acquired ultrasound images using dedicated post-processing software integrated into the institutional imaging workflow.

Vascularization patterns were assessed using the Lagalla classification system, which categorizes intranodular vascularity according to progressive grades from absent vascular flow to marked central vascularization [11]. The evaluation was performed by a single experienced operator (G.A.) before treatment and during follow-up examinations.

Post-ablation ultrasound assessment focused on progressive changes in nodule volume, vascular distribution, and structural appearance of the treated area. Reduction or disappearance of intranodular vascular signals was considered an indicator of effective thermal injury and subsequent tissue remodeling [12,13,14,15].

**Figure 3:**
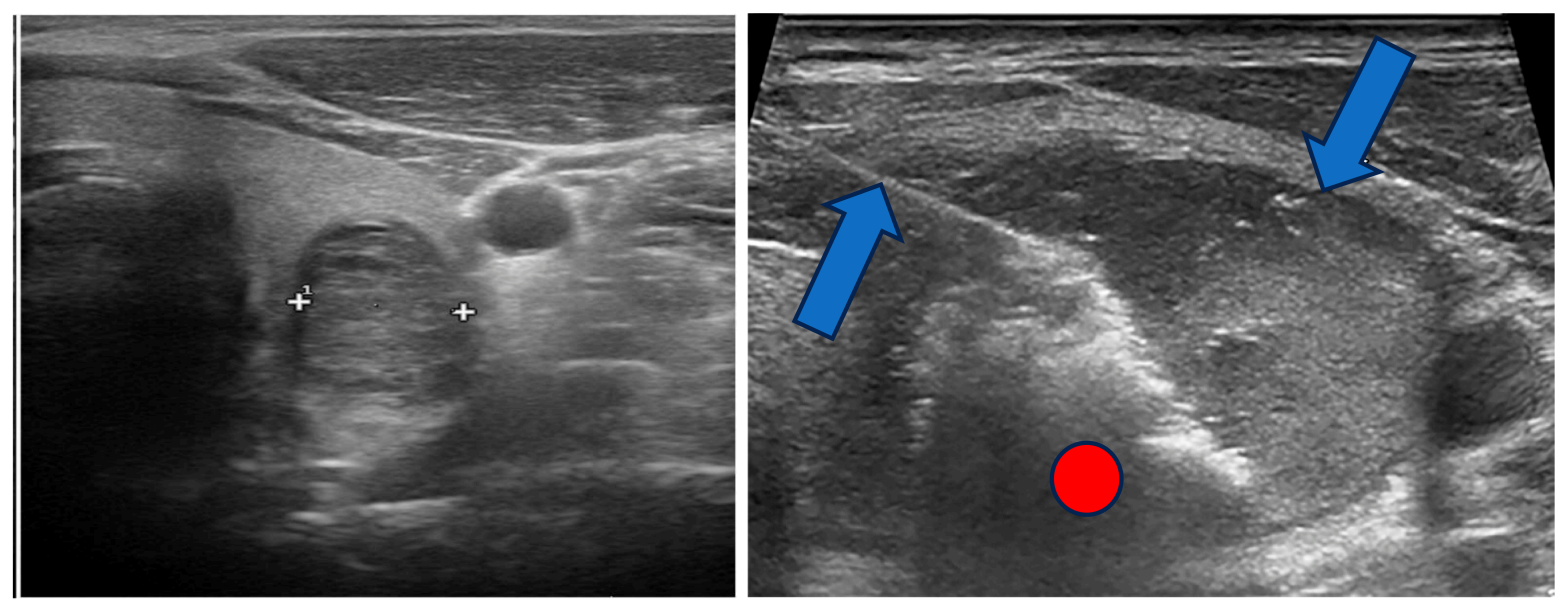
High-resolution ultrasound evaluation before and after radiofrequency ablation. Images were obtained from the institutional ultrasound database (AOU Luigi Vanvitelli). The blue line on the left indicates the ablation needle entering the nodule; the blue line on the right indicates the hypoechoic nodule to be treated, separated from the surrounding tissue; the red dot indicates the hyperechoic area, signifying that the thermoablation treatment has taken place.

Technical success was defined as complete coverage of the target nodule by the ablation zone immediately after treatment, confirmed by ultrasound evaluation. Clinical effectiveness was assessed through volumetric reduction, improvement of compressive symptoms, and cosmetic outcome during follow-up (Figure 3).

Post-ablation vascular changes were interpreted according to the known biological mechanisms of thermal injury, including coagulative necrosis, microvascular thrombosis, and progressive fibrotic remodeling of treated tissue [16,17,18].

This imaging protocol was consistent with previously described standardized ultrasound approaches for thyroid nodule evaluation and post-ablation monitoring, which emphasize the integration of morphological, vascular, and volumetric parameters for treatment response assessment [7,19,20].

### 2.4. Radiofrequency Ablation Technique

Ultrasound-guided radiofrequency ablation (RFA) was performed in all enrolled patients according to a standardized institutional protocol and in accordance with international recommendations for thyroid thermal ablation procedures [6,21].

All procedures were performed by a single operator (G.A.) with expertise in thyroid ultrasonography and ultrasound-guided interventional procedures. Patients were placed in a supine position with mild neck extension to optimize exposure of the cervical region. The procedure was performed under sterile conditions using local anesthesia with 1% lidocaine infiltrated at the skin puncture site and along the perithyroidal space. Conscious sedation was not routinely required.

A trans-isthmic approach was used whenever feasible to ensure a stable electrode trajectory and minimize the risk of thermal injury to adjacent critical structures, including the recurrent laryngeal nerve, trachea, and esophagus [21]. Radiofrequency ablation was performed using a VIVA RF generator (Starmed, Seoul, Republic of Korea) equipped with an internally cooled monopolar electrode with a 10 mm active tip.

**Figure 4:**
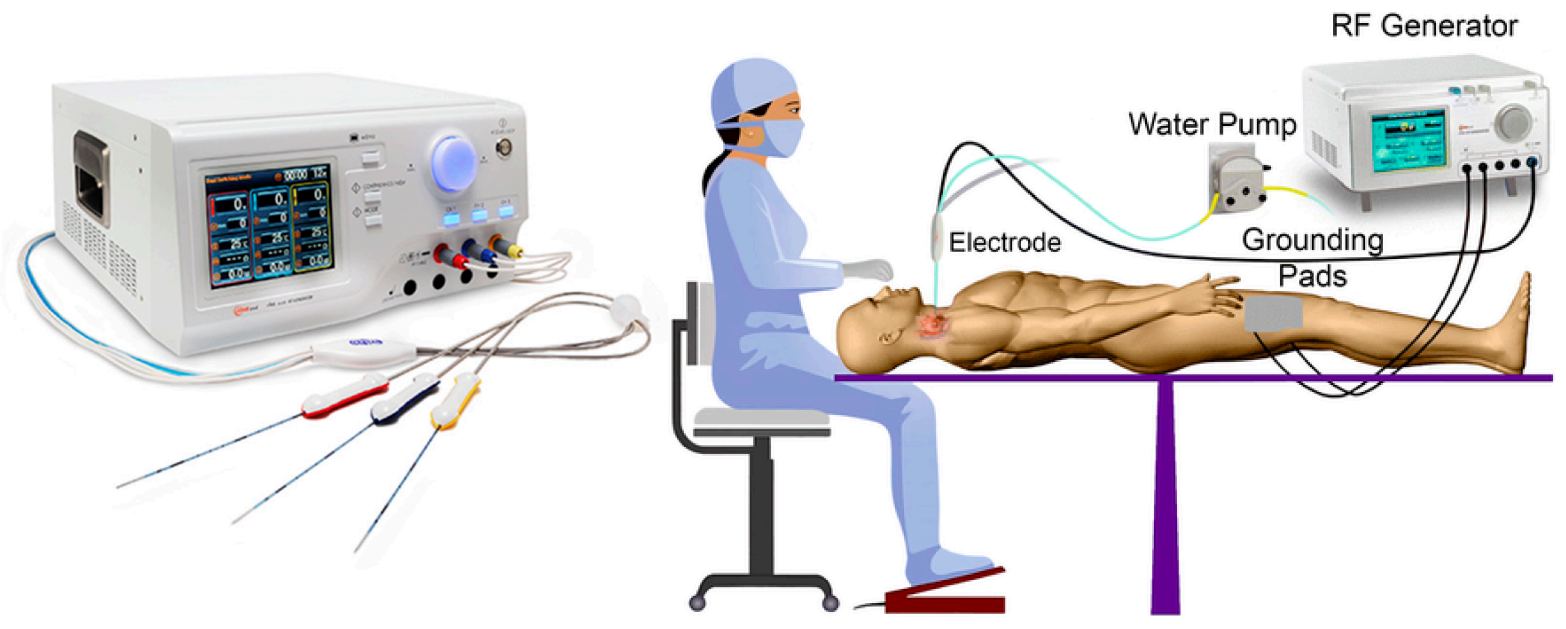
Radiofrequency ablation technique [6,21].

A standardized moving-shot technique was applied, with progressive repositioning of the electrode within different regions of the nodule to achieve homogeneous thermal coverage. Ablation was initiated from the deepest portion of the lesion and progressively extended toward superficial areas under continuous ultrasound guidance, according to previously validated thyroid RFA protocols [20,21] (Figure 4).

Power output was adjusted according to nodule size, tissue impedance changes, and ultrasound visualization of the ablation zone. The mean applied power in the study cohort was 30 W, with a mean ablation time of approximately 8 min per treated nodule. The electrode was repositioned multiple times within the target lesion according to the moving-shot technique until complete coverage of the nodule was achieved.

The procedural endpoint was defined as complete transformation of the treated nodule into a hyperechoic ablation zone extending over the entire target lesion, with adequate safety margins whenever anatomically feasible. Additional ablation was performed in cases of incomplete coverage or persistence of residual vascular signals within the treated area.

Hydrodissection with saline solution was performed when necessary to create a protective thermal barrier between the ablation zone and adjacent vulnerable structures, particularly in nodules located near the trachea, carotid sheath, or recurrent laryngeal nerve.

Immediate post-procedural ultrasound examination was performed to evaluate the treated area and exclude early complications. Patients were monitored for at least one hour after the procedure and discharged if clinically stable. Procedure-related adverse events were recorded, including voice changes suggestive of recurrent laryngeal nerve injury, hematoma, skin burns, and other local complications.

Treatment response was assessed during follow-up examinations at 1, 3, 6, and 12 months, including ultrasound evaluation, volumetric analysis, symptom assessment, and documentation of clinical outcomes.

### 2.5. Molecular and Extracellular Vesicle (EV) Analysis

Peripheral blood samples were collected from all patients before radiofrequency ablation and prior to any interventional procedure. Blood collection was performed under standardized conditions, and samples were immediately processed for plasma separation according to established protocols for extracellular vesicle (EV) studies [22,23].

EVs were isolated from plasma samples using a combination of differential centrifugation and size-exclusion chromatography, a methodology selected to improve EV enrichment while reducing contamination from cellular debris and circulating protein aggregates [22]. EV characterization was performed according to the recommendations of the Minimal Information for Studies of Extracellular Vesicles (MISEV) guidelines, including evaluation of particle concentration and size distribution through nanoparticle tracking analysis (NTA) [23].

Total RNA was extracted from EV-enriched fractions, including EV-associated microRNAs (miRNAs). Exploratory miRNA profiling was performed using a microarray-based hybridization approach to identify circulating miRNA patterns potentially associated with thyroid nodule characteristics and tissue response following thermal ablation.

Raw molecular data were processed through quality-control procedures, including assessment of signal intensity, exclusion of low-quality probes, and normalization of expression values before integrative analysis. Differential expression analysis and correlation analyses were performed in an exploratory manner to investigate potential associations between EV-derived miRNA profiles, imaging parameters, and post-ablation tissue remodeling features.

**Figure 5:**
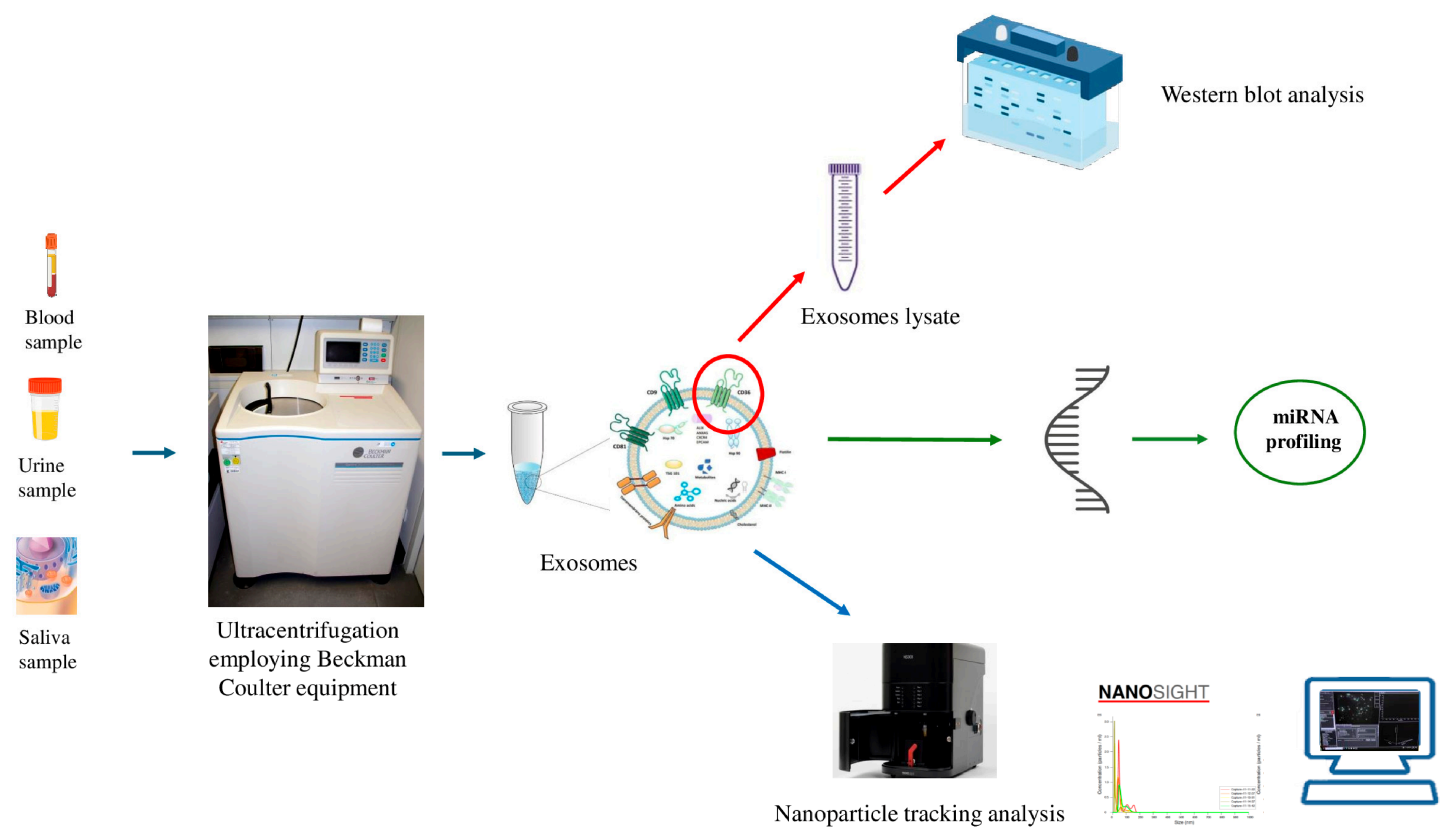
Molecular and extracellular vesicle analysis. Images were obtained from the institutional database. (AOU Luigi Vanvitelli).

The rationale for investigating EV-derived miRNAs was based on their recognized role as stable circulating mediators involved in cellular communication, inflammatory responses, tissue remodeling, and disease-related biological processes [24,25]. In the present study, molecular profiling was considered exploratory and hypothesis-generating, aiming to integrate circulating molecular information with clinical, imaging, and histopathological datasets within a translational framework (Figure 5).

### 2.6. Multimodal Integration, Surgical Validation and Translational Framework

All clinical, imaging, molecular, and histopathological data were integrated within a multimodal translational framework designed to investigate tissue response following ultrasound-guided radiofrequency ablation (RFA).

The framework combined four complementary data domains: (1) clinical parameters, including demographic characteristics, symptoms, thyroid function, and treatment outcomes; (2) ultrasound-derived parameters, including nodule volume, volumetric reduction rate, vascularization pattern changes, and three-dimensional reconstruction data; (3) extracellular vesicle (EV)-derived microRNA profiles obtained from peripheral blood samples before treatment; and (4) histopathological findings obtained from patients who subsequently underwent thyroidectomy.

A dedicated integrative workflow was used to align longitudinal clinical and imaging data with molecular and tissue-level information. Ultrasound datasets were analyzed together with EV-derived molecular profiles to explore potential associations between baseline biological characteristics and post-ablation response patterns.

Patients who developed indications for surgery during follow-up underwent thyroidectomy according to standard clinical criteria. The indications for surgery included insufficient volumetric response after RFA, defined as inadequate nodule reduction during follow-up evaluation, associated with persistent clinical relevance of the treated lesion.

In the surgical validation subgroup, thyroidectomy specimens were analyzed by conventional histopathology and digital pathology reconstruction. Histological evaluation focused on treatment-related tissue changes, including coagulative necrosis, fibrosis, inflammatory remodeling, vascular alterations, and the presence of residual viable thyroid tissue within or adjacent to the treated area.

The multimodal framework enabled spatial and biological correlation between pre-treatment ultrasound characteristics, circulating EV-derived molecular profiles, and post-ablation tissue architecture. The aim of this integrative approach was exploratory, providing a translational model for the characterization of RFA-induced tissue remodeling and for the future development of personalized response prediction strategies.

**Figure 6:**
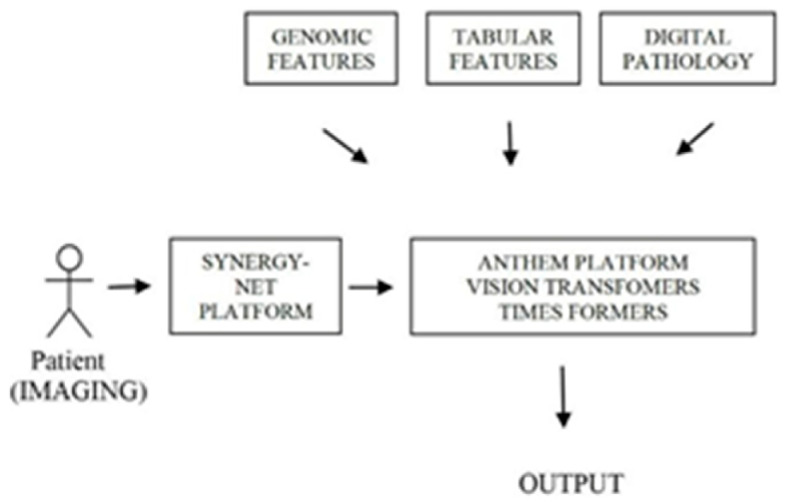
Intelligent platform for integration of clinical, imaging, molecular, and histopathological data.

This workflow represents a proof-of-concept approach integrating imaging biomarkers, liquid biopsy-derived molecular information, and pathological validation within a precision medicine framework (Figure 6).

### 2.7. Statistical Analysis

Statistical analyses were performed using IBM SPSS Statistics software, version 30.0 (IBM Corp., Armonk, NY, USA). Continuous variables were assessed for distribution using the Shapiro–Wilk test and were reported as mean ± standard deviation (SD) for normally distributed variables or as median with interquartile range (IQR) for non-normally distributed variables. Categorical variables were expressed as absolute numbers and percentages.

Changes in thyroid nodule volume during follow-up were evaluated by comparing baseline values with post-treatment measurements using paired statistical tests according to data distribution. For normally distributed variables, the paired Student’s *t*-test was applied, whereas the Wilcoxon signed-rank test was used for non-parametric comparisons.

The volume reduction rate (VRR) was calculated for each patient, and longitudinal changes at different follow-up time points were evaluated using repeated-measures analysis when appropriate. Differences between subgroups were assessed using the independent Student’s *t*-test or Mann–Whitney U test for continuous variables and the χ^2^ test or Fisher’s exact test for categorical variables.

Associations between clinical variables, ultrasound parameters, and treatment outcomes were explored using correlation analyses. A two-sided *p*-value < 0.05 was considered statistically significant.

Patients with incomplete follow-up evaluations were included in the analysis according to available data. Missing values were not imputed due to the observational and exploratory nature of the study.

For extracellular vesicle-derived microRNA analysis, molecular comparisons were considered exploratory. Expression data were normalized before analysis, and differential expression patterns were investigated using appropriate bioinformatic approaches. Associations between miRNA expression profiles, imaging characteristics, and clinical outcomes were explored without considering the molecular analysis as a definitive biomarker validation study. Due to the exploratory nature of molecular profiling and the limited sample size, findings were interpreted as hypothesis-generating and requiring external validation.

## 3. Results

### 3.1. Nodule Volume Reduction

In the overall cohort of 60 patients, ultrasound-guided radiofrequency ablation (RFA) resulted in a progressive reduction in thyroid nodule volume during follow-up.

The mean baseline nodule volume was 39.8 mL. At the 2-month evaluation, the mean volume reduction rate (VRR) was 56%, corresponding to a significant decrease in treated nodule volume compared with baseline measurements (*p* < 0.001).

Serial ultrasound examinations performed at 1, 3, 6, and 12 months demonstrated a progressive and sustained reduction in nodule size over time. The reduction in volume was associated with progressive morphological changes in the treated area, including reduction in intranodular vascularity and increasing fibrotic remodeling on ultrasound evaluation.

**Figure 7:**
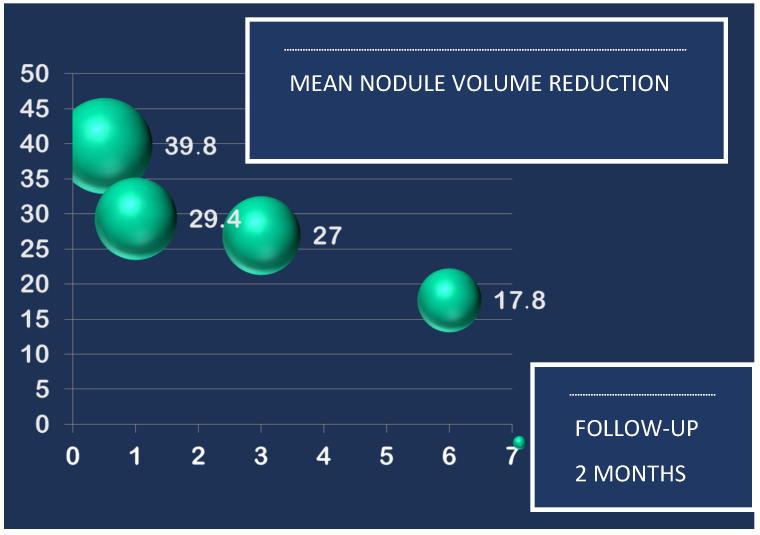
Volume reduction. The mean nodule volume reduction was 56% as evaluated at the 2-month follow-up.

The volumetric response was heterogeneous among patients, reflecting differences in baseline nodule characteristics, vascular pattern, and biological response to thermal injury. However, the majority of treated nodules showed clinically relevant volume reduction during follow-up (Figure 7).

The volume reduction rate was calculated for each patient according to the following formula (2):

VRR (%) = [(initial volume − final volume)/initial volume] × 100.(2)

In the overall cohort of 60 patients, baseline nodule volume was 39.8±14.2 mL (median: 36.5 mL, IQR: 28.0–48.2 mL). At the 2-month evaluation, mean nodule volume decreased to 17.5±8.1 mL, achieving a mean volume reduction rate (VRR) of 56.0±12.4% (p<0.001, paired t-test). At 6-month and 12-month evaluations, mean VRR reached 64.2±11.8% (p<0.001) and 68.5±13.1% (p<0.001), respectively.

### 3.2. Symptom Relief and Cosmetic Outcomes

Clinical evaluation demonstrated a progressive improvement in compressive symptoms and cosmetic concerns after radiofrequency ablation.

At baseline, symptoms related to local compression, including cervical discomfort, swallowing impairment, and pressure sensation, were reported in a substantial proportion of patients. During follow-up, a progressive reduction in symptom burden was observed, with an overall improvement of approximately 44% compared with baseline clinical assessment.

The reduction in compressive symptoms was statistically significant when comparing pre-treatment and post-treatment evaluations (*p* < 0.001).

Cosmetic outcomes also improved progressively during follow-up. Nodules initially classified as visibly deforming or clearly visible showed a progressive reduction in external appearance, with a shift toward lesions classified as barely visible or not visible after treatment.

The improvement in cosmetic appearance was consistent with the volumetric reduction observed on ultrasound evaluation and reflected the progressive shrinkage and remodeling of the treated nodules.

**Figure 8:**
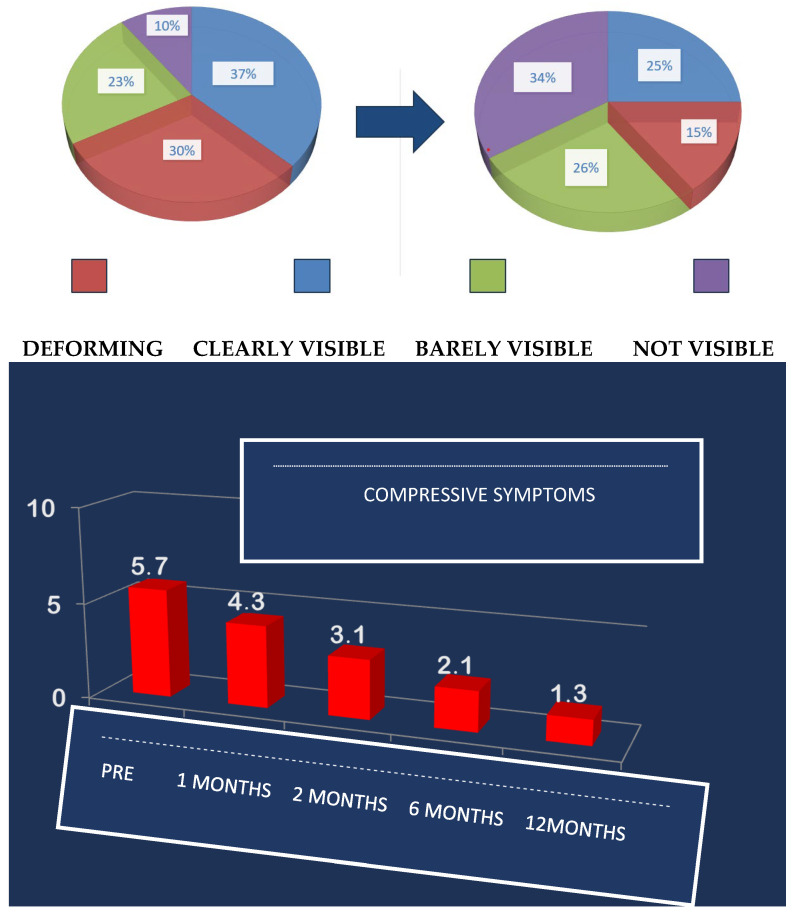
Clinical and cosmetic improvement. From a prevalence of deforming and clearly visible lesions, we obtained a prevalence of barely visible and not visible lesions. The reduction in compressive symptoms was 44%.

Clinical outcomes were assessed together with ultrasound findings, including volume reduction and vascular pattern changes, in order to provide an integrated evaluation of treatment effectiveness (Figure 8).

### 3.3. Vascularization Pattern Changes

**Figure 9:**
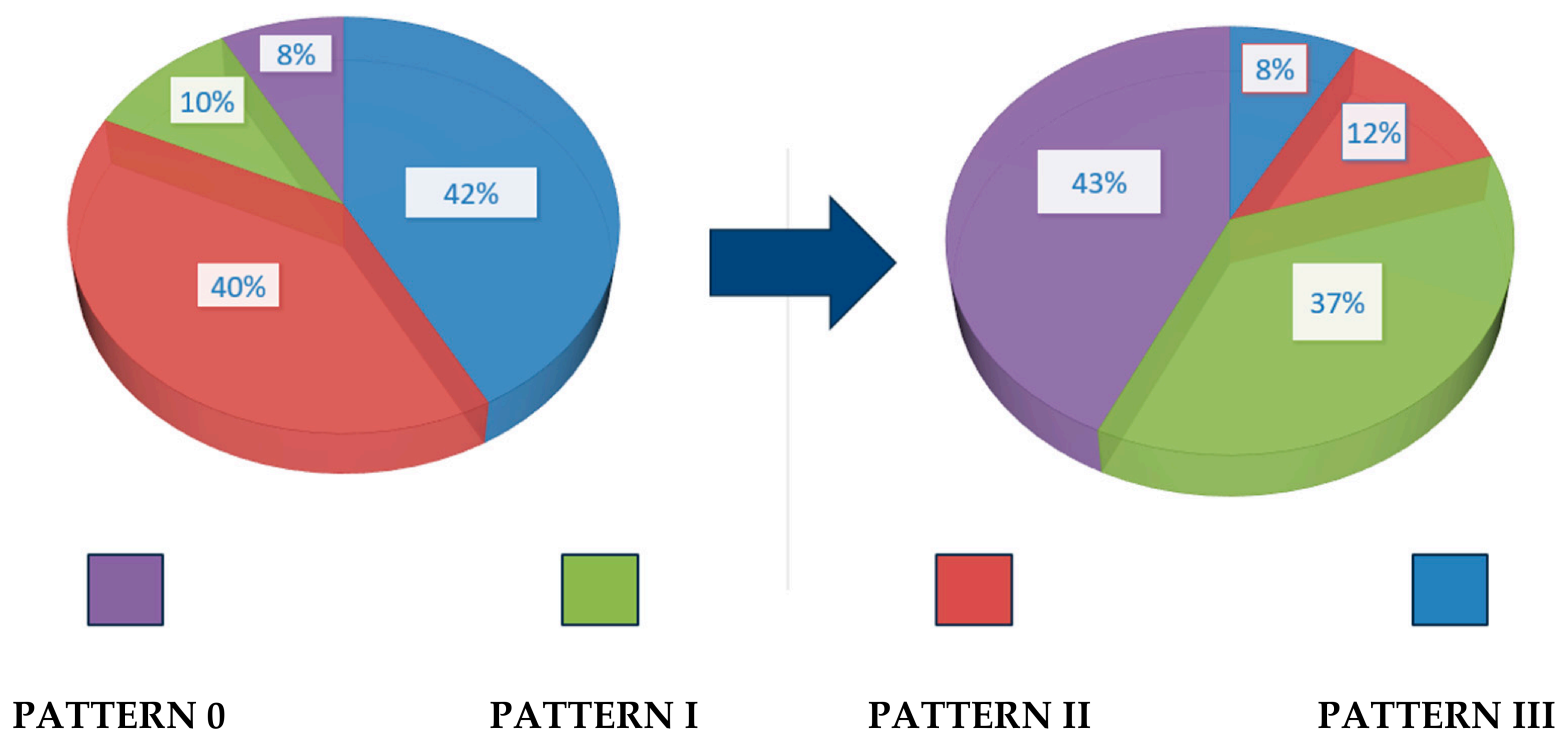
Vascularization pattern changes. From a prevalence of “Pattern II” and “Pattern III” (Lagalla Score), we obtained a prevalence of “Pattern 0” and “Pattern.

Color Doppler ultrasound evaluation using the Lagalla classification system demonstrated a significant post-procedural shift in intranodular vascularity (*p* < 0.001). At baseline, the majority of nodules exhibited high to intermediate vascularization: 38 nodules (63.3%) were classified as Pattern III (marked central and peripheral vascularization) and 18 nodules (30.0%) as Pattern II (moderate vascularization), while only 4 nodules (6.7%) presented Pattern I (peripheral flow only). During longitudinal follow-up, thermal ablation induced progressive devascularization. At the 6-month follow-up evaluation, a marked shift toward avascular or low-vascularity patterns was documented: 35 nodules (58.3%) were reclassified as Pattern 0 (complete absence of intranodular flow) and 19 nodules (31.7%) as Pattern I. Only 6 nodules (10.0%) maintained persistent Pattern II/III vascularity, which correlated with incomplete ablation areas. This progressive devascularization directly reflected acute microvascular thrombosis followed by fibrotic tissue remodeling (Figure 9).

All the results are shown in Table 2.

### 3.4. Surgical Subgroup and Histopathological Validation

**Figure 10:**
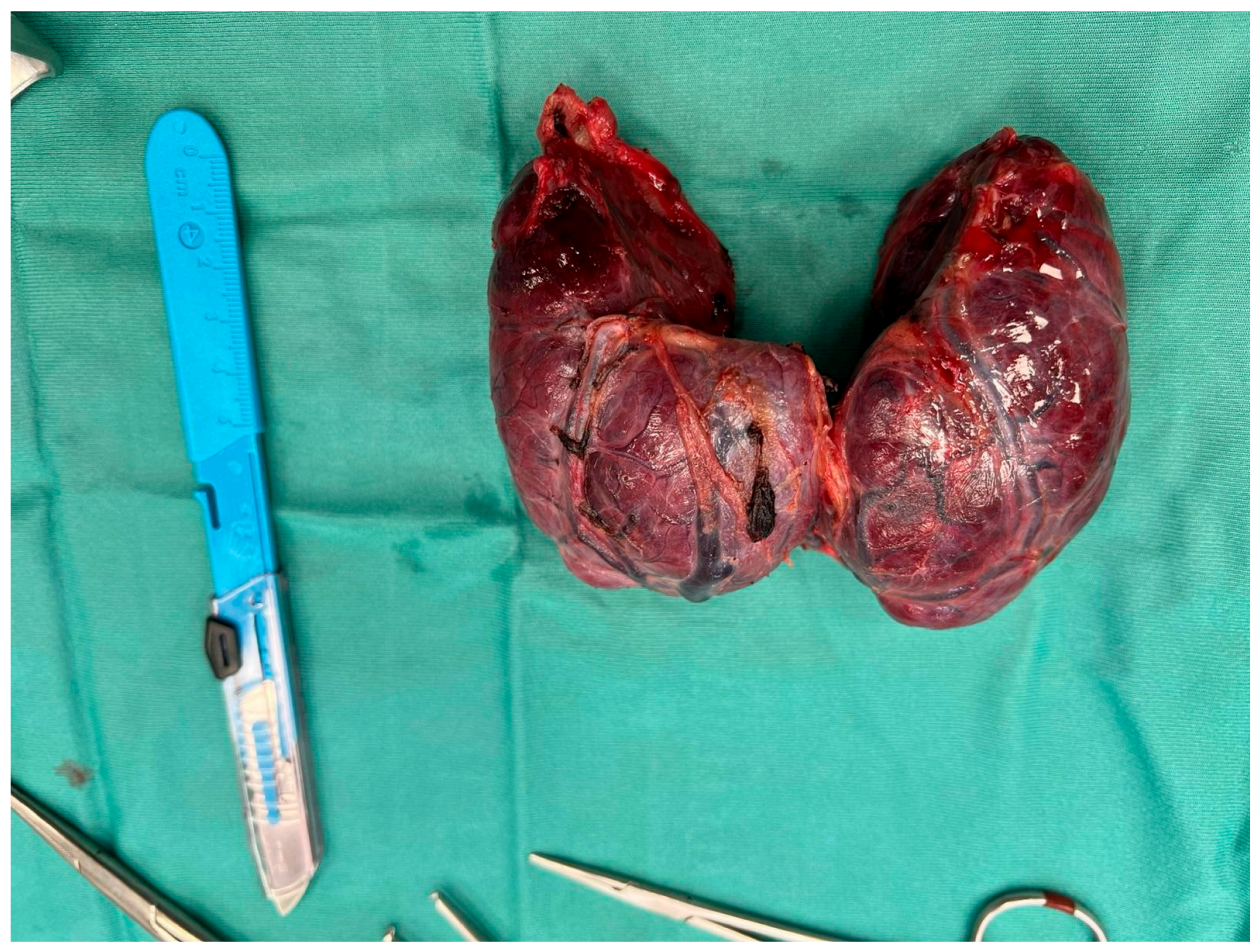
A subset of patients who developed subsequent surgical indications underwent thyroidectomy after RFA treatment. In this image, you can see a total thyroidectomy specimen including both thyroid lobes and the isthmus, submitted for histopathological examination.

**Figure 11:**
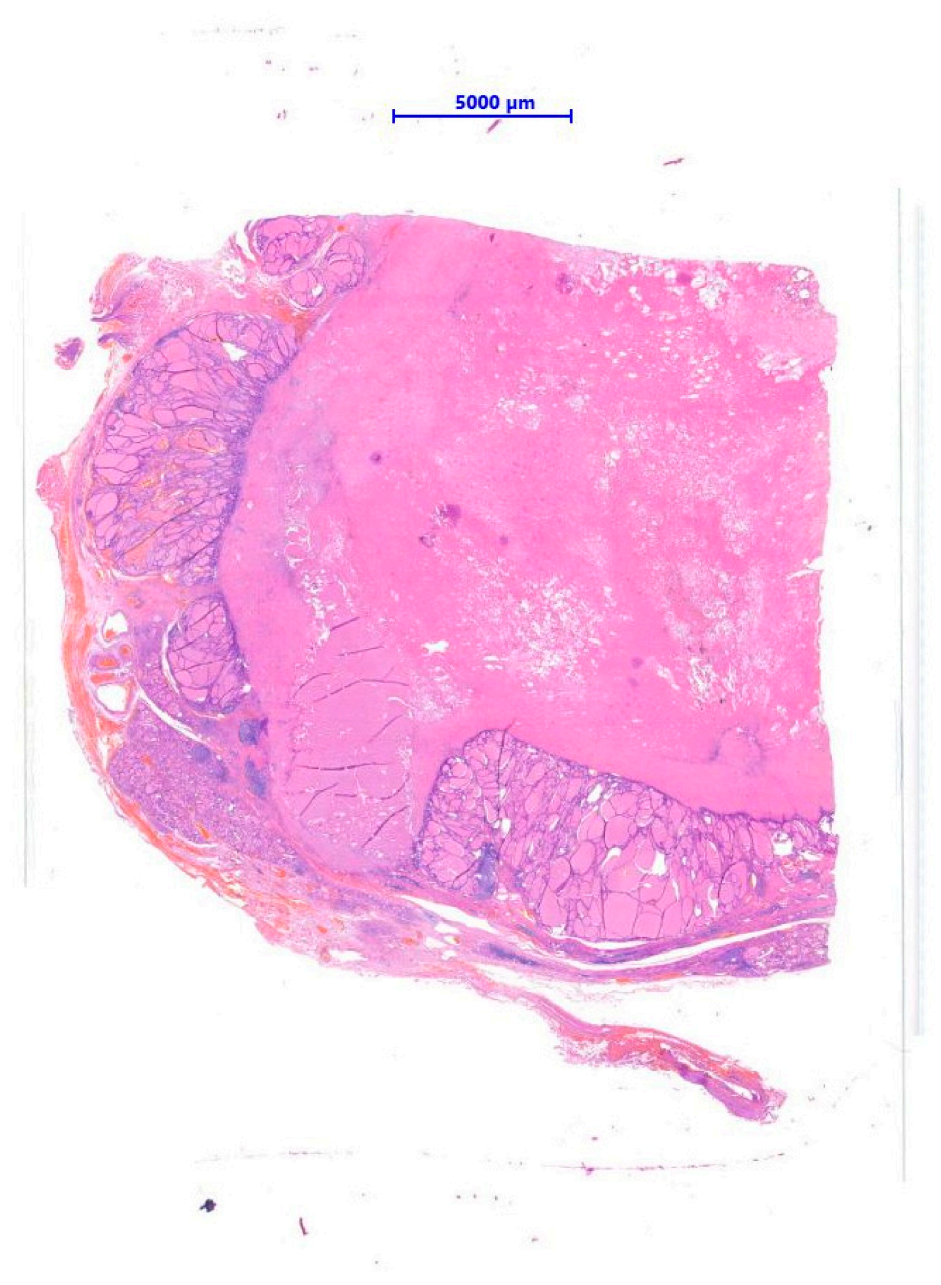
In this image, you can clearly see the nodule that underwent the thermo-ablation procedure surrounded by a normally functioning thyroid. Images were obtained from the institutional database. (AOU Luigi Vanvitelli).

**Figure 12:**
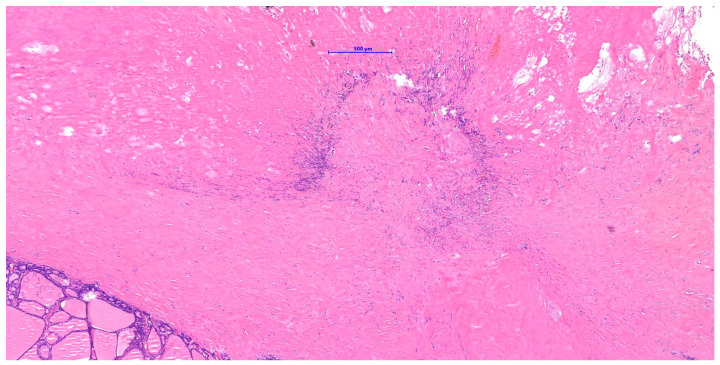
In this area, residual fibrosis from the procedure is observed, associated with chronic inflammatory infiltrate. Images were obtained from the institutional database. (AOU Luigi Vanvitelli).

**Figure 13:**
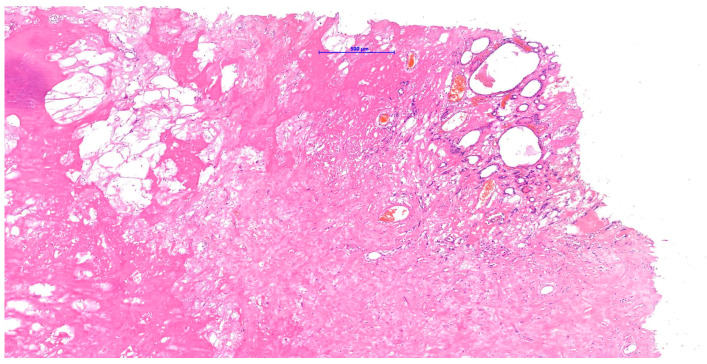
Necrosis and residual thyroid follicles. Images were obtained from the institutional database. (AOU Luigi Vanvitelli).

**Figure 14:**
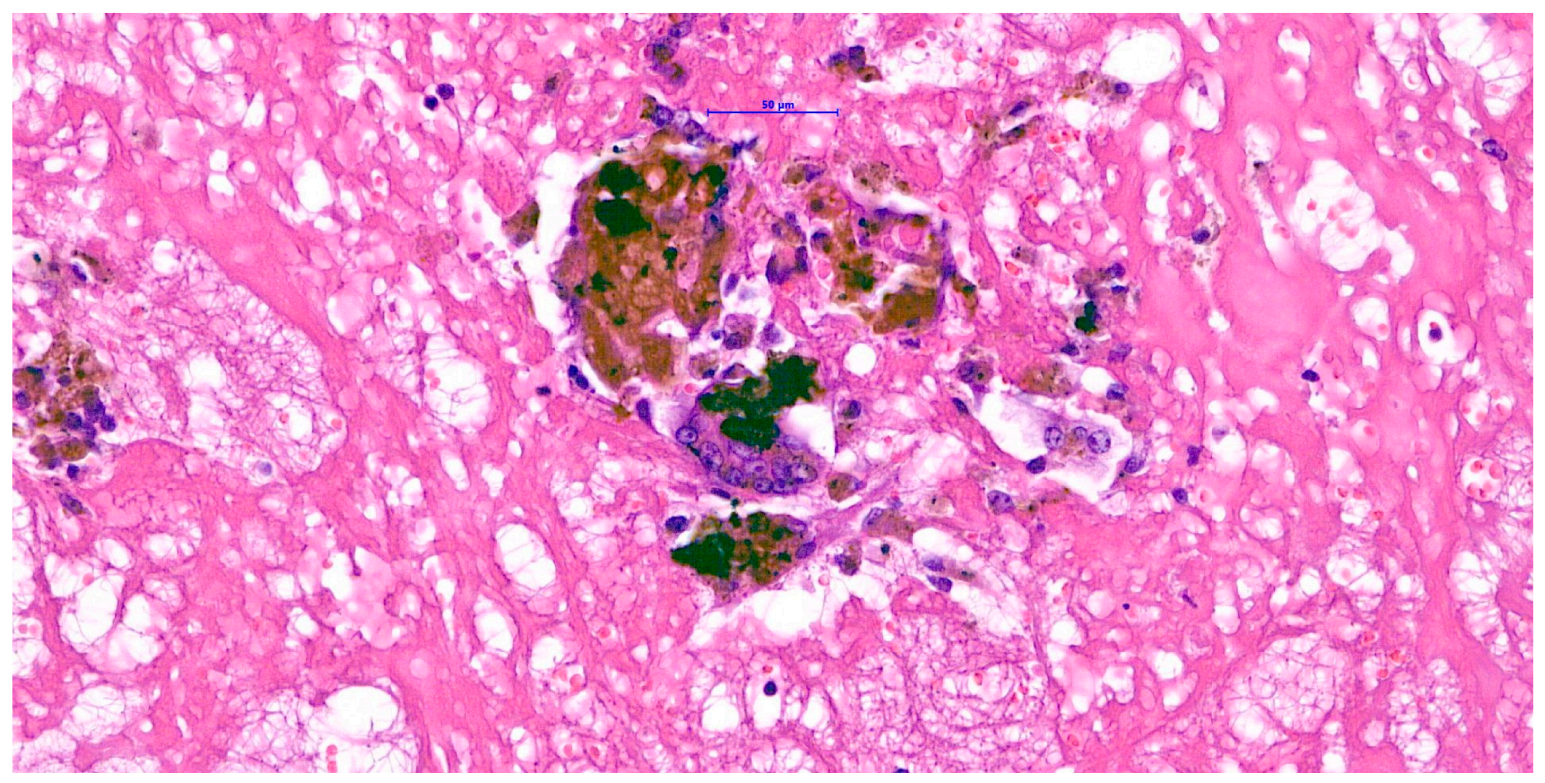
In an area with fibrosis and necrosis, there is accumulation of hemosiderin with a characteristic multinucleated giant cell phagocytosing hemosiderin. Images were obtained from the institutional database. (AOU Luigi Vanvitelli).

A subgroup of 10 patients (16.7% of the total cohort; 8 females, 2 males; mean age: 48.5 ± 9.2 years) underwent total or hemithyroidectomy during the follow-up period. The primary indication for surgery was insufficient volumetric response (defined as a volume reduction rate < 50% at 6 months) associated with persistent compressive symptoms (*n* = 7) or cosmetic dissatisfaction and patient preference (*n* = 3). The median time interval between RFA treatment and subsequent surgery was 6.5 months (interquartile range [IQR]: 4.0–9.0 months; total range: 3–12 months). Histopathological evaluation of resected surgical specimens confirmed treatment-induced structural alterations and provided direct tissue validation of the imaging findings. Macroscopic examination revealed well-demarcated nodular lesions containing central necrotic zones surrounded by a fibrous capsule (Figure 9 and Figure 10). Microscopic analysis demonstrated characteristic thermal injury hallmarks:

- Central Zone: Extensive coagulative necrosis characterized by ghost-like cellular outlines, loss of follicular architecture, and pyknotic nuclei.
- Intermediate/Peripheral Zone: Dense fibrotic tissue deposition, chronic inflammatory infiltrate composed of lymphocytes and plasma cells, and microvascular thrombosis (Figure 11 and Figure 12).
- Foreign-Body Reaction: Areas of hemorrhage showing marked hemosiderin deposition, accompanied by hemosiderin-laden macrophages and characteristic multinucleated giant cells engaged in phagocytosis (Figure 13).
- Rim Zone: Variable islands of residual viable thyroid follicles were predominantly observed at the outer periphery of the ablation zone, accounting for incomplete volumetric shrinkage in this subgroup (Figure 14).

Digital pathology reconstruction confirmed the spatial heterogeneity of tissue response, demonstrating high topographical concordance with pre-surgical ultrasound color Doppler maps.

### 3.5. Molecular (EV-miRNA) Findings and Translational Correlations

Quality control evaluation of isolated extracellular vesicles (EVs) confirmed successful enrichment according to MISEV guidelines, displaying a characteristic particle size distribution (mean diameter: 118.4 ± 14.2 nm) and expected particle concentrations. Exploratory microarray hybridization of baseline circulating EV-derived miRNAs identified differential expression profiles among patients with varying degrees of therapeutic response. Using threshold criteria of an absolute fold change (|FC|) > 1.5 and an adjusted *p*-value < 0.05, a distinct subset of candidate microRNAs associated with post-ablation tissue response was identified:

- Pro-inflammatory and Fibrotic Correlates: Baseline circulating levels of miR-21-5p and miR-146b-5p were significantly upregulated in patients achieving high volume reduction rates (VRR ≥ 60% at 6 months; *p* = 0.003 and *p* = 0.012, respectively), consistent with their known roles in driving acute tissue inflammation and subsequent fibrotic remodeling;
- Non-Responder Molecular Profile: Conversely, elevated baseline levels of miR-221-3p and miR-222-3p correlated negatively with 6-month volumetric response (Spearman r = −0.42, *p* = 0.008 and r = −0.38, *p* = 0.016, respectively).

Patients in the surgical subgroup (who exhibited insufficient volumetric response, VRR < 50%) demonstrated significantly higher pre-procedural EV-miR-221-3p expression compared to optimal responders (*p* = 0.004). These exploratory molecular findings suggest that baseline circulating EV-miRNA profiles may reflect intrinsic tissue susceptibility to thermal injury and post-ablation tissue remodeling (Figure 5).

### 3.6. Multimodal Translational Integration

To combine heterogeneous data domains into a standardized predictive architecture, a computational multimodal integrative framework was implemented. The analytical pipeline processed four distinct data layers:

- Clinical Layer: Demographic parameters, baseline symptom scores, and thyroid functional status.
- Imaging Layer: 3D ultrasound volumetric measurements, nodule echotexture metrics, and Doppler Lagalla vascular scores.
- Molecular Layer: Normalized baseline EV-miRNA expression matrices (focusing on candidate markers miR-21-5p, miR-146b-5p, miR-221-3p, and miR-222-3p).
- Histopathology Layer: Digital pathology quantitative metrics (percentage of coagulative necrosis, fibrotic area, and peripheral residual viable thyroid parenchyma) in the surgical subgroup.

Data integration was executed using a supervised feature-fusion workflow. Machine learning algorithms (including Random Forest feature selection and Logistic Regression models) were explored to assess the feasibility of pre-treatment response stratification. The multimodal integrative model achieved a preliminary area under the receiver operating characteristic curve (AUC-ROC) of 0.81 (95% CI: 0.69–0.93) for discriminating high responders (VRR ≥ 60%) from poor responders (VRR < 50%). While these computational results serve as a translational proof-of-concept, the platform currently functions as an analytical research framework rather than an automated clinical decision tool, requiring further prospective validation in larger, multi-center cohorts (Figure 6).

## 4. Discussion and Conclusions

In this prospective observational study, ultrasound-guided radiofrequency ablation (RFA) demonstrated high clinical efficacy and safety in the treatment of benign and autonomously functioning thyroid nodules, achieving a mean volume reduction rate of 56% at early follow-up and a 44% improvement in compressive symptoms (p<0.001) without major complications [26,27,28,29] aligning closely with the established international literature reporting volume reductions between 50% and 80% [1,2,6,20,30]. Beyond clinical outcomes, Color Doppler ultrasound analysis revealed a significant post-ablation shift from Lagalla patterns II–III to 0–I (p<0.001), reflecting acute microvascular thrombosis and progressive devascularization [11,16]. Crucially, histopathological evaluation of the 10 patients (16.7% of the total cohort) who subsequently underwent thyroidectomy due to insufficient volumetric response (volume reduction rate <50%) provided direct tissue validation, demonstrating central coagulative necrosis, extensive fibrotic deposition, and chronic inflammation with hemosiderin-laden giant cells, alongside peripheral viable follicles that account for incomplete volumetric shrinkage in this subgroup [30]. To address the current lack of tissue-level biomarkers, exploratory circulating extracellular vesicle (EV)-derived miRNA profiling revealed candidate molecular signatures—specifically upregulation of miR-21-5p and miR-146b-5p in optimal responders versus elevated miR-221-3p and miR-222-3p in non-responders—supporting the concept that circulating EVs reflect localized tissue stress and remodeling [24,25,31]; however, given the exploratory nature and lack of secondary cohort validation, these molecular findings must be interpreted cautiously as hypothesis-generating. Furthermore, integrating clinical, 3D ultrasound, miRNA, and histopathological datasets within a computational framework yielded a proof-of-concept model for response stratification (AUC = 0.81), though its current role remains an analytical research tool rather than an automated clinical decision system [32,33,34]. Several limitations must be acknowledged, including the monocentric observational design, the modest overall sample size without a formal a priori power calculation or a non-treated/surgical control group, the limited size of the surgical subgroup, and the medium-term follow-up duration (mean 11 months). In conclusion, RFA is confirmed as a safe and effective minimally invasive procedure for benign symptomatic thyroid nodules, and while further multi-center validation is required, this multimodal translational framework offers a novel integrative perspective bridging clinical phenotype, radiological dynamics, and underlying molecular biology toward personalized patient management.

## Figures and Tables

**Table 1:** Guideline-based indications for thyroid radiofrequency ablation (RFA) [6,7,8].

Society/Guideline	Indications	Key Criteria	Main Limitations
European Thyroid Association (2020) [6]	Benign symptomatic thyroid nodules; selected autonomously functioning thyroid nodules (AFTN)	Confirmed benign cytology (Bethesda II); compressive symptoms; cosmetic concern; AFTN refractory to medical therapy	No integration of molecular or tissue biomarkers
Korean Society of Thyroid Radiology (KSThR) (2017- Updated consensus) [7]	Benign solid or predominantly solid nodules; AFTN	Ultrasound benign features + cytology; symptom/cosmetic scoring; suppressed TSH in AFTN	Limited biological stratification; operator-dependent outcomes
American Thyroid Association (2015 ATA Guidelines for Thyroid Nodules) [8]	Indirect support for minimally invasive approaches in benign nodules	Risk stratification based on ultrasound + cytology	No specific recommendation for thermal ablation techniques

**Table 2:** Summary of Clinical, Volumetric, and Vascular Outcomes Before and After Radiofrequency Ablation (RFA).

Parameter	Baseline (*n* = 60)	Follow-Up/Post-RFA	Magnitude of Effect	*p*-Value
**Nodule** **Volume (mL), mean ± SD (median [IQR])**	39.8 ± 14.2 (36.5 [28.0–48.2])	17.5 ± 8.1 (15.2 [11.0–22.4]) at 2 months	Mean Volume Reduction Rate (VRR): 56.0 ± 12.4%	*p* < 0.001
**Compressive** **Symptom Score (0–10 VAS), mean ± SD**	6.8 ± 1.8	3.8 ± 1.4 (at 6 months)	44.1% reduction in symptom burden	*p* < 0.001
**Vascular** **Pattern (Lagalla),** ***n* (%)**	-	-	Significant shift toward devascularization	*p* < 0.001
**Pattern 0** **(Absent)**	0 (0%)	35 (58.3%) at 6 months	+58.3%	
**Pattern I** **(Peripheral)**	4 (6.7%)	19 (31.7%) at 6 months	+25.0%	
**Pattern II (Moderate)**	18 (30%)	4 (6.7%) at 6 months	−23.3%	
**Pattern III (Marked)**	38 (63.3%)	2 (3.3%) at 6 months	−60%	
**Cosmetic score, *n* (%)**			Shift to non/barely visible	*p* < 0.001
**Deforming/Clearly** **Visible**	52 (86.7%)	11 (18.3%) at 6 months	−68.4%	
**Barely/Not Visible**	8 (13.3%)	49 (81.7%) at 6 months	+68.4%	

## Data Availability

The original contributions presented in this study are included in the article. Further inquiries can be directed to the corresponding author.

## References

[1] Issa P.P., Cironi K., Rezvani L., Kandil E. (2024). Radiofrequency ablation of thyroid nodules: A clinical review of treatment complications. Gland. Surg..

[2] Muhammad H., Santhanam P., Russell J.O. (2021). Radiofrequency ablation and thyroid nodules: Updated systematic review. Endocrine.

[3] Trimboli P., Castellana M., Sconfienza L.M., Virili C., Pescatori L.C., Cesareo R., Giorgino F., Negro R., Giovanella L., Mauri G. (2020). Efficacy of thermal ablation in benign non-functioning solid thyroid nodule: A systematic review and meta-analysis. Endocrine.

[4] Baek J.H., Jeong S.Y., Feingold K.R., Adler R.A., Ahmed S.F., Anawalt B., Blackman M.R., Chrousos G., Corpas E., de Herder W.W., Dhatariya K., Dungan K. (2000). Thermal Ablation for Thyroid Nodules. Endotext [Internet].

[5] Ha E.J., Lee M.K., Baek J.H., Lim H.K., Ahn H.S., Baek S.M., Choi Y.J., Chung S.R., Kim J.H., Shin J.H. (2025). Radiofrequency Ablation for Recurrent Thyroid Cancers: 2025 Korean Society of Thyroid Radiology Guideline. Korean J. Radiol..

[6] Papini E., Monpeyssen H., Frasoldati A., Hegedüs L. (2020). 2020 European Thyroid Association Clinical Practice Guideline for the Use of Image-Guided Ablation in Benign Thyroid Nodules. Eur. Thyroid. J..

[7] Kim J.H., Baek J.H., Lim H.K., Ahn H.S., Baek S.M., Choi Y.J., Choi Y.J., Chung S.R., Ha E.J., Hahn S.Y. (2018). 2017 Thyroid Radiofrequency Ablation Guideline: Korean Society of Thyroid Radiology. Korean J. Radiol..

[8] Haugen B.R. (2017). 2015 American Thyroid Association Management Guidelines for Adult Patients with Thyroid Nodules and Differentiated Thyroid Cancer: What is new and what has changed?. Cancer.

[9] Muhammad H., Santhanam P., Russell J.O., Kuo J.H. (2021). RFA and benign thyroid nodules: Review of the current literature. Laryngoscope Investig. Otolaryngol..

[10] Ha E.J., Baek J.H., Che Y., Chou Y.H., Fukunari N., Kim J.H., Lin W.C., My L.T., Na D.G., Quek L.H.H. (2021). Radiofrequency ablation of benign thyroid nodules: Recommendations from the Asian Conference on Tumor Ablation Task Force. Ultrasonography.

[11] Yang G.C.H., Fried K.O. (2017). Most Thyroid Cancers Detected by Sonography Lack Intranodular Vascularity on Color Doppler Imaging: Review of the Literature and Sonographic-Pathologic Correlations for 698 Thyroid Neoplasms. J. Ultrasound Med..

[12] Cece A., Agresti M., De Falco N., Sperlongano P., Moccia G., Luongo P., Miele F., Allaria A., Torelli F., Bassi P. (2025). Role of Artificial Intelligence in Thyroid Cancer Diagnosis. J. Clin. Med..

[13] De Falco N., Agresti M., De Falco M., Sperlongano P., Moccia G., Luongo P., Cece A., Bove F., Miele F., Allaria A. (2025). Simultaneous Medullary and Papillary Thyroid Carcinomas: Personal Experience Report and Literature Review. J. Clin. Med..

[14] Parmeggiani D., Cece A., Agresti M., Miele F., Luongo P., Moccia G., Torelli F., Sperlongano R., Bassi P., Savabi Far M. (2026). Improved Multisource Image-Based Diagnostic for Thyroid Cancer Detection: ANTHEM National Complementary Plan Research Project. Appl. Sci..

[15] Dobrinja C., Samardzic N., Giudici F., Raffaelli M., De Crea C., Sessa L., Docimo G., Ansaldo G.L., Minuto M., Varaldo E. (2021). Hemithyroidectomy versus total thyroidectomy in the intermediate-risk differentiated thyroid cancer: The Italian Societies of Endocrine Surgeons and Surgical Oncology Multicentric Study. Updates Surg..

[16] Russ G., Bonnema S.J., Erdogan M.F., Durante C., Ngu R., Leenhardt L. (2017). European Thyroid Association Guidelines for Ultrasound Malignancy Risk Stratification of Thyroid Nodules in Adults: The EU-TIRADS. Eur. Thyroid. J..

[17] Frates M.C., Benson C.B., Charboneau J.W., Cibas E.S., Clark O.H., Coleman B.G., Cronan J.J., Doubilet P.M., Evans D.B., Goellner J.R. (2005). Management of thyroid nodules detected at US: Society of Radiologists in Ultrasound consensus conference statement. Radiology.

[18] Li S., Yu M.A., Zhao Z.L., Wei Y., Peng L.L., Li Y. (2025). Changes in thyroid function after thermal ablation of thyroid nodules. Front. Endocrinol..

[19] Kim H.J., Baek J.H., Cho W., Sim J.S. (2022). Long-term follow-up of the radiofrequency ablation of benign thyroid nodules: The value of additional treatment. Ultrasonography.

[20] Mauri G., Orsi F., Carriero S., Della Vigna P., De Fiori E., Monzani D., Pravettoni G., Grosso E., Manzoni M.F., Ansarin M. (2021). Image-Guided Thermal Ablation as an Alternative to Surgery for Papillary Thyroid Microcarcinoma: Preliminary Results of an Italian Experience. Front. Endocrinol..

[21] Huber T.C., Park A.W. (2021). Radiofrequency Ablation of Benign Thyroid Nodules. Semin. Interv. Radiol..

[22] Théry C., Witwer K.W., Aikawa E., Alcaraz M.J., Anderson J.D., Andriantsitohaina R., Antoniou A., Arab T., Archer F., Atkin-Smith G.K. (2018). Minimal information for studies of extracellular vesicles 2018 (MISEV2018): A position statement of the International Society for Extracellular Vesicles and update of the MISEV2014 guidelines. J. Extracell. Vesicles.

[23] Kalluri R., LeBleu V.S. (2020). The biology, function, and biomedical applications of exosomes. Science.

[24] Valadi H., Ekström K., Bossios A., Sjöstrand M., Lee J.J., Lötvall J.O. (2007). Exosome-mediated transfer of mRNAs and microRNAs is a novel mechanism of genetic exchange between cells. Nat. Cell Biol..

[25] Mitchell P.S., Parkin R.K., Kroh E.M., Fritz B.R., Wyman S.K., Pogosova-Agadjanyan E.L., Peterson A., Noteboom J., O’Briant K.C., Allen A. (2008). Circulating microRNAs as stable blood-based markers for cancer detection. Proc. Natl. Acad. Sci. USA.

[26] Collins F.S., Varmus H. (2015). A new initiative on precision medicine. N. Engl. J. Med..

[27] Weissleder R., Pittet M.J. (2008). Imaging in the era of molecular oncology. Nature.

[28] Esteva A., Robicquet A., Ramsundar B., Kuleshov V., DePristo M., Chou K., Cui C., Corrado G., Thrun S., Dean J. (2019). A guide to deep learning in healthcare. Nat. Med..

[29] Kourou K., Exarchos T.P., Exarchos K.P., Karamouzis M.V., Fotiadis D.I. (2014). Machine learning applications in cancer prognosis and prediction. Comput. Struct. Biotechnol. J..

[30] Dobrinja C., Bernardi S., Fabris B., Eramo R., Makovac P., Bazzocchi G., Piscopello L., Barro E., de Manzini N., Bonazza D. (2015). Surgical and Pathological Changes after Radiofrequency Ablation of Thyroid Nodules. Int. J. Endocrinol..

[31] Sim J.S., Baek J.H. (2019). Long-Term Outcomes Following Thermal Ablation of Benign Thyroid Nodules as an Alternative to Surgery: The Importance of Controlling Regrowth. Endocrinol. Metab..

[32] Yu F., Moehring A., Banerjee O., Salz T., Agarwal N., Rajpurkar P. (2024). Heterogeneity and predictors of the effects of AI assistance on radiologists. Nat. Med..

[33] Lambin P., Leijenaar R.T.H., Deist T.M., Peerlings J., de Jong E.E.C., van Timmeren J., Sanduleanu S., Larue R.T.H.M., Even A.J.G., Jochems A. (2017). Radiomics: The bridge between medical imaging and personalized medicine. Nat. Rev. Clin. Oncol..

[34] Orloff L.A., Noel J.E., Stack B.C., Russell M.D., Angelos P., Baek J.H., Brumund K.T., Chiang F., Cunnane M.B., Davies L. (2022). Radiofrequency ablation and related ultrasound-guided ablation technologies for treatment of benign and malignant thyroid disease: An international multidisciplinary consensus statement of the American Head and Neck Society Endocrine Surgery Section with the Asia Pacific Society of Thyroid Surgery, Associazione Medici Endocrinologi, British Association of Endocrine and Thyroid Surgeons, European Thyroid Association, Italian Society of Endocrine Surgery Units, Korean Society of Thyroid Radiology, Latin American Thyroid Society, and Thyroid Nodules Therapies Association. Head Neck.

